# A commentary of “The brain evolutionary mechanism of feeding preference”: 10 remarkable discoveries from 2020 in *Nature*

**DOI:** 10.1016/j.fmre.2022.01.010

**Published:** 2022-01-31

**Authors:** Yufeng Pan

**Affiliations:** The Key Laboratory of Developmental Genes and Human Disease, School of Life Science and Technology, Southeast University, Nanjing, 210096, China

## Abstract

The fly *Drosophila sechellia* feeds exclusively on the toxic noni fruit (*Morinda citrifolia*). What makes this species such a picky eater compared with its generalist relatives? Auer et al. cracked the case using the genome-editing tool CRISPR/Cas9 [1]. One sensory neuron class expressing the odorant receptor 22a (Or22a) protein is more abundant in *D. sechellia* than in other fly species. The group established that small changes in Or22a's amino-acid sequence have contributed to *D. sechellia*’s preference for noni. They also identified several other evolutionary changes that might contribute to this apparently simple behavioral shift. Even tiny flies that love stinky fruit can provide powerful insight into how brains evolve to shape complex behaviors.①

The “10 remarkable discoveries from 2020” listed in the journal *Nature* includes a basic research article on the feeding behavior of fruit flies, which is surprisingly refreshing. Why was the secret of fruit fly feeding selected as one of the top ten scientific discoveries of the year?

Animal behaviors undergo adaptive changes in specific natural environments. Studying the mechanism of these behavioral changes helps to deepen our understanding of how animal behaviors, including human behaviors, are generated and modulated. Although the theory of evolution is well known, the mechanism of behavioral evolution has rarely been studied in-depth, as it requires such study on how a certain behavior evolves not only in a model animal species but also in other closely related species.

*Drosophila melanogaster* is a classic animal model for studying behavioral regulation mechanisms. For example, the molecular mechanism of the biological clock that earned the 2017 Nobel Prize in Physiology or Medicine was discovered using this fruit fly model. In recent years, the broad application of CRISPR/Cas9 gene-editing technology has made it possible to apply various genetic tools previously used only in the model organism *D. melanogaster* to other *Drosophila* species, especially those with their genomes already sequenced. This provides an ideal animal model for in-depth investigation of how the behaviors of two or more closely related species evolve. Using feeding behavior as an example, one species of fruit fly (*D. sechellia*) has a unique feeding preference: it feeds primarily on a poisonous, foul-smelling rotten fruit (*Morinda citrifolia*, commonly known as noni or vomit fruit), while other closely related *Drosophila* species, including *D. melanogaster*, feed on more common fruits (such as bananas and grapes). The unique feeding preference of *D. sechellia* may be derived from the abundance of noni and the lack of other fruits on the Seychelles Islands in the Indian Ocean hundreds of thousands of years ago, which drove the evolution of nervous and metabolic systems unique to noni feeding in this species.

A team led by Dr. Richard Benton at the University of Lausanne in Switzerland systematically compared the feeding preferences of three fruit fly species (*D. melanogaster, D. sechellia*, and *D. simulans*). They generated a large number of mutant flies and other genetic tools simultaneously in these three fruit fly species and revealed the evolutionary mechanisms of feeding preference. First, they identified the olfactory receptor protein Or22a that is necessary for perceiving the smell of noni in different fruit fly species, and the Or22a-expressing neurons that receive and transmit such olfactory information. Second, the Or22a orthologues in *D. sechellia* and *D. melanogaster* are almost identical, but the change of several amino acids of Or22a, especially one particular amino acid, from the *D. melanogaster* version to the *D. sechellia* version, and vice versa, is sufficient to at least partially reverse the noni odor response in the two species, revealing the primary molecular mechanism in the evolution of this behavior. Third, *D. sechellia* not only evolved a form of the Or22a protein that is more sensitive to the odor of noni, but it also has about twice as many sensory neurons expressing this protein compared to other *Drosophila* species. Finally, central neurons in *D. sechellia* that process olfactory information have also undergone changes in their projection, which may change their response to the smell from repulsion to attraction. This study reveals that the evolution of feeding preference is manifested at multiple scales: from the mutation of a single amino acid in the olfactory receptor protein to the change in the number of neurons expressing the receptor protein, to the changes in the projections of central neurons that integrate sensory stimuli.

The most remarkable feature of this study is the depth and breadth of its research on behavioral evolution mechanisms. The researchers established a large number of genetic tools in the three *Drosophila* species at the same time, studied the regulation of feeding behavior of each species in parallel in both the sensory system and the central nervous system, and investigated the evolutionary mechanism of feeding preference to the single-gene and single-neuron resolution. Considerable progress has been made in research on the mechanisms of animal behavior in recent years, including the outstanding contributions of many Chinese researchers to dissect the regulatory mechanisms of instinctive behavior, learning and memory, forgetting, depression, and autism. However, these studies are often limited to a specific animal model and could answer the question of “how is the behavior regulated?” in that particular species. Dr. Benton's work will stimulate more research on the mechanisms of behavioral evolution between different species to solve the less-investigated questions such as “how does the behavior evolve?” and “why did the behavior evolve in this way?”

## Declaration of Competing Interest

The author declares that he has no conflicts of interest in this work.
